# Patients presenting at the emergency department with acute abdominal pain are less likely to be admitted to inpatient wards at times of access block: a registry study

**DOI:** 10.1186/s13049-015-0158-3

**Published:** 2015-10-07

**Authors:** MC Blom, M. Landin–Olsson, M. Lindsten, F. Jonsson, K. Ivarsson

**Affiliations:** 1 Department of Clinical Sciences Lund, Lund University, HS 32, EA-blocket, 2nd floor, SE-22185 Lund, Sweden; 2 Department of Surgery, Ystad General Hospital, Kristianstadsvägen 3A, SE-27182 Ystad, Sweden; 3 Department of Pre- and Intrahospital Emergency Medicine, Helsingborg General Hospital, S Vallgatan 5, SE-25187 Helsingborg, Sweden

**Keywords:** [MeSH]: Emergency medicine, Emergency medicine organization and administration, Acute abdominal pain, Bed occupancy, Emergency department overcrowding

## Abstract

**Background:**

Also known as access block, shortage of inpatient beds is a common cause of emergency department (ED) boarding and overcrowding, which are both associated with impaired quality of care. Recent studies have suggested that access block not simply causes boarding in EDs, but may also result in that patients are less likely to be admitted to the hospital from the ED. The present study’s aim was to investigate whether this effect remained for patients with acute abdominal pain, for which different management strategies have emerged. Access block was defined in terms of hospital occupancy and the appropriateness of ED discharges addressed as 72 h revisits to the ED.

**Methods:**

As a registry study of ED administrative data, the study examined a population of patients who presented with acute abdominal pain at the ED of a 420-bed hospital in southern Sweden during 2011–2013. Associations between exposure and outcomes were addressed in contingency tables and by logistic regression models.

**Results:**

Crude analysis revealed a negative association between access block and the probability of inpatient admission (38.6 % admitted at 0–95 % occupancy, 37.8 % at 95–100 % occupancy, and 35.0 % at ≥100 % occupancy) (*p* < .001). No significant associations between exposure and 72 h revisits emerged. Multivariable models indicated an odds ratio of inpatient admission of 0.992 (95 % CI: 0.986–0.997) per percentage increase in hospital occupancy.

**Conclusions:**

Study findings indicate that patients with acute abdominal pain are less likely to be admitted to the hospital from the ED at times of access block and that other management strategies are employed instead. No association with 72 h revisits was seen, but future studies need to address more granular outcomes in order to clarify the safety aspects of the effect.

**Electronic supplementary material:**

The online version of this article (doi:10.1186/s13049-015-0158-3) contains supplementary material, which is available to authorized users.

## Background

Shortage of inpatient beds—that is, access block or “hospital crowding”—is a prominent cause of emergency department (ED) boarding [1, 2] and overcrowding [1–7]. Both effects are associated with impaired quality of care [2], the latter often for causing treatment delays [9–11], increased mortality [11–13], and patient dissatisfaction [14, 15]. Recent studies have suggested that hospital crowding not only causes boarding in the ED, but also that ED patients are less likely to be admitted to the hospital at times of access block and instead are discharged home [16]. Such admission-bias may reflect a strategy by which ED staff averts inpatient admission in all but the sickest patients [16, 17]. Patients with acute abdominal pain frequently seek care in the ED and different management strategies have emerged for those lacking immediate indications for surgical treatment [18]. One strategy is to admit them to the hospital and make them subject to early laparoscopy (EL) [19–24], or close observation [18–20, 25]. Another strategy is to use radiology in order to rule out time-sensitive conditions [26–31], sometimes without admitting the patient to hospital.

The study is primarily hypothesis-generating, aiming at evaluating whether the management strategy for ED patients with acute abdominal pain changes as a function of hospital bed-availability, so that patients become less likely to be admitted to the hospital at times of access block. The appropriateness of ED discharges is addressed by the 72 h revisit rate. A secondary aim is to compare the ED length of stay (EDLOS) across different levels of access block, in discharged patients.

## Methods

### Study design

For this registry study of ED administrative data, the sample consisted of patients who presented with a primary complaint of abdominal pain at the surgical and emergency medicine (EM) specialty units in the ED of a 420-bed hospital in southern Sweden during 2011–2013. Presentations at these facilities were selected in order to exclude other causes of abdominal pain, such as those assessed at the internal medicine specialty unit (e.g. pyelonephritis, gastroenteritis). Patients less than 18 years of age, who died in the ED, who left the ED against medical advice, and/or who were transferred to another hospital were also excluded.

### Setting

The ED of Helsingborg General Hospital serves a population of roughly 250,000, which expands to more than 300,000 in the summer. It is one of four emergency hospitals in the region of Skåne in southern Sweden. The annual ED census of physician visits shows an increase from just below 60,000 to 65,000 from 2011 to 2013. Upon arrival, all patients were registered by secretaries in the information system Patientliggaren^®^. The approximately 15 % of patients who arrived by ambulance, or who have been referred to the ED by a physician—typically from primary care—gained access to the ED directly after registration. Other patients gained access to the ED in accordance with predefined guidelines or were further evaluated by a nurse in primary triage. Primary triage refers to a sorting-facility where decisions are made as to whether a patient should be cared for in the ED or be referred to another level of care (discharged home, primary care). After being admitted to the ED, patients underwent secondary triage (an algorithm for prioritizing ED patients depending on vital parameters and main complaints, resembling what is used in most EDs worldwide).

Secondary triage was performed by a trained triage-nurse, using a five-level triage system implemented in 2013 and known as the rapid emergency triage and treatment system (RETTS^©^) [32], though during its validation period was called the medical emergency triage and treatment system (METTS) [33]. One of the five levels of RETTS^©^ signifies no indication for emergency care and was often assigned to patients referred to another level of care by primary triage. Patient triage category was registered in Patientliggaren^®^ by the nurse who performed secondary triage.

After secondary triage, patients were directed to separate units for surgery, orthopedics, medicine, and otolaryngology in a triage-to-specialty model [34]. A complementary unit staffed by emergency physicians capable of addressing various complaints except for psychiatric, otolaryngologic, ophthalmologic, and pediatric (medicine) ones was introduced in 2010 that operated from 8 am to 11 pm daily. In late 2012, this facility assumed increased responsibility for surgical patients. There are separate EDs for children with medical conditions (<18 years of age) and for patients with obstetric/gynecologic, psychiatric, or ophthalmologic complaints. Visits to these EDs were excluded from this study, as were patients less than 18 years of age assessed at the surgical or EM facility. Patients transferred from the surgical/EM facilities to another facility who did not return—most were transferred to the obstetric/gynecologic facility and there received final assessment—were also excluded, though patients who received their final assessment at the surgical/EM facilities after transferring from another facility were included. Consequently, cases that were deemed to most likely suffer from a surgical condition by the triage-nurse, but where an attending physician made a different assessment, were excluded. In the case of a scheduled revisit to the ED, physical ED records from the index visit were stored at each specialty desk, and triage nurses made notes in Patientliggaren^®^ upon patient arrival. Radiology and laboratory analyses were available to ED patients around the clock. Some ED physicians perform bedside US in the ED, but the general rule is that patients who need radiology were referred to the radiology department. Since the clinical observation unit was introduced in late 2012, patients admitted there have been considered admitted to the hospital for billing purposes and are treated as such in the present study.

### Sample size

*Post hoc* power calculations were performed to determine cutoff levels for strata of in-hospital bed occupancy to use in crude comparisons for α = 0.05 and 80 % power (1–β = 0.80) [35]. Differences of 3 % for inpatient admission, 2 % for 72 h revisits, and 1 % for 72 h revisits resulting in admission were specified as clinically relevant *a priori* to analysis. Ten events per predictor were considered adequate for multivariable analysis [36].

### Data sources

Data regarding patient visits were retrieved from the ED information system Patientliggaren^®^. Data concerning hourly occupancy levels were obtained from the hospital informatics unit and extracted by a professional data manager. The datasets were merged by an author (MB) in the programming language Python™.

### Variables

Access block was defined in terms of hospital occupancy (the number of occupied beds in the hospital divided by the number of staffed beds) at the beginning of the hour when the patient presented at the ED. The total occupancy for somatic wards (i.e. non-psychiatric wards) accepting patients from the ED (i.e. not exclusively surgical wards) was used because of the full-capacity protocols that took effect during hospital crowding, thereby causing patients admitted from the ED to be distributed evenly among wards sorting under different departments. Sample size calculations revealed that the study material was sufficient for applying a three-category variable (<95 %, 95–100 %, ≥100 %) indicating access block in the crude analysis, though only a dichotomous variable was acceptable for evaluating 72 h revisits and ED length of stay (EDLOS). Since 95 % reflects the median occupancy at the hospital, <95 % was used a common-sense reference. In the case of the dichotomous variable, the cutoff of 100 % occupancy was preferred to that of 95 %, since median occupancy may not reflect true access block. Inpatient admission was indicated in Patientliggaren^®^ as a dichotomous variable. Unplanned 72 h revisits were defined as revisits within 72 h of the initial visit, to the study site or to the nearby ED of Ängelholm General Hospital, and that were not identified as planned revisits in Patientliggaren^®^. Sex, triage category, and high ED input were all coded as dichotomous variables. The triage dichotomy reflected medical urgency (i.e., priority 1 and 2 patients were considered time sensitive as they needed to be seen by a physician within 15 min). High ED input was indicated by the 75^th^ percentile of shifts receiving most ED visits (adjusted for time of week). Time of year (Dec–Feb and Jun–Aug versus the rest), time of week (Mon and Sat–Sun versus the rest), and shift (00:00–08:00, 08:00–16:00, and 16:00–00:00) were constructed as three-level categorical variables.

### Crude analysis

Fisher’s exact test was applied to compare crude proportions of outcomes across levels of occupancy. The Mann–Whitney U test was used to compare EDLOS across strata of in-hospital bed occupancy for patients not admitted to an inpatient ward during their index visit. Due to the recent controversy regarding applying non-parametric tests to non-normal data in large datasets [37], their parametric counterparts were used for comparison.

### Multivariable analysis

Logistic regression was used to adjust for any confounders and covariates in multivariable analyses of the association of access block with inpatient admission and 72 h revisits. Directed acyclic graphs were used to identify the appropriate set of independent variables for adjustment [38, 39]. Causal models were developed by all authors using the free online tool “DAGitty” (Additional files 1 and 2) [40]. The minimally sufficient adjustment set for addressing all three outcomes consisted of time of year, time of week, and shift (time of day). The adjustment set was entered into the logistic equation using the entry method instead of a stepwise method. Interaction terms included were based on empirical knowledge and comprised occupancy*shift. Independent variables of the minimally sufficient adjustment set not significantly associated with the outcome were retained to prevent bias [38, 39]. Interaction terms of weaker association with the outcome than *p* = .05 upon inclusion in the model were omitted.

The adequacy of expected cell counts was assessed in contingency tables [41], while multicollinearity was addressed by variance inflation factor (VIF) and tolerance statistics. Age and in-hospital bed occupancy were screened for linearity in the logit using the Box–Tidwell approach [41] and, if violated, were transformed to the ordinal scale. Multivariable outliers were addressed by Mahalanobis distance and evaluated according to an *X* ^2^ distribution at *p* = .001 [42]. To improve the face validity of the multivariable models, sensitivity analysis was performed by expanding the minimally sufficient adjustment set (step 1) in two subsequent steps (steps 2, 3). In step 2, triage category, age, and sex were added to the list of covariates. In step 3, three variables—the first indicating whether the patient entered the ED via primary triage, the second indicating whether ED input was high during the simultaneous shift, and the third indicating year 2013, which captures the introduction of RETTS^©^, increased responsibility for surgical patients at the EM facility, and the introduction of an observation unit—were added to the list of covariates. The selection of variables for expansion was based on knowledge of risk factors for admission in the present dataset [16] and the possibility of a wider spectrum of underlying disease in females suffering from abdominal pain.

Sensitivity analyses for 72 h revisits excluded triage priority, whether the patient entered the ED via primary triage, and whether the patient presented during a shift with high input. This was because these parameters were only recorded during the index visit and therefore were not considered relevant to the situation during the revisit. The model’s goodness-of-fit was evaluated with the likelihood ratio test, and effect size was evaluated with Nagelkerke’s *R* ^*2*^. The likelihood ratio and Wald tests were used to evaluate the contribution of individual variables, and model dispersion parameters were used to rescale the Wald statistic appropriately [42]. Standardized residuals were used to identify influential cases. Since a total of three multivariable models and two crude comparisons were developed for each outcome, Bonferroni adjustment was applied to yield significance at *p* = .01. Statistical analyses were performed in the Statistical Package for the Social Sciences^®^ version 22 (IBM). The Regional Ethical Review Board in Lund granted ethical approval for the study (dnr 2013/11).

## Results

### Participants

**Fig. 1:**
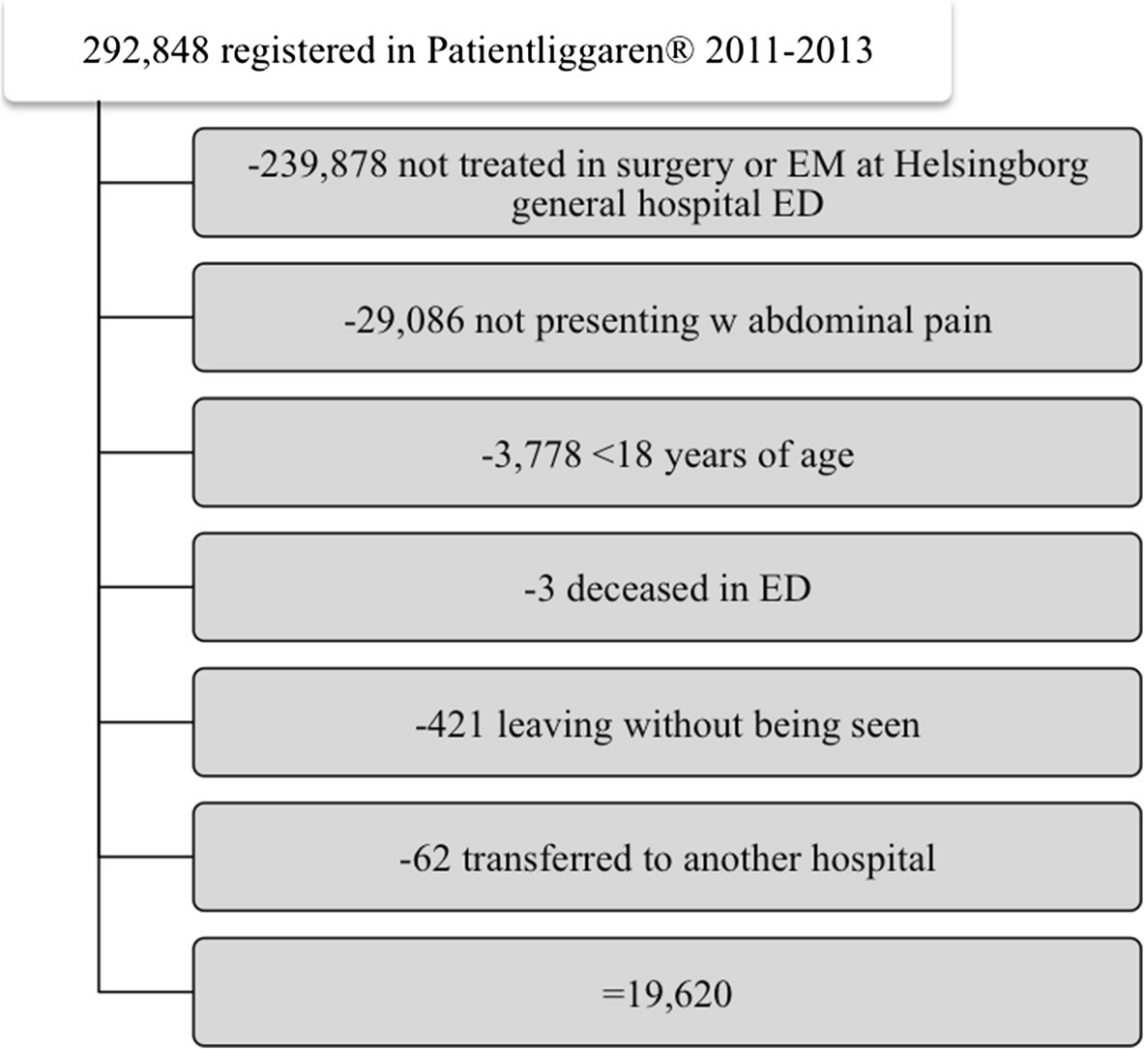
Exclusion analysis

In all, 52,970 visits were made to the EM and surgical facilities of the ED at Helsingborg General Hospital during 2011–2013. Of these visits, 23,884 cases presented with a primary complaint of abdominal pain, 3,778 of which were less than 18 years of age and thus excluded, along with three patients who died in the ED, 421 who left against medical advice, and 62 who were transferred to another hospital. The final study population was thus 19,620 cases (Fig. 1).

### Missing data

Missing data appeared only in the variable indicating triage category (83/19,620 = 0.4 %). Since no verified predictors of triage priority were present in the dataset, regression imputation was not feasible, which in conjunction with their scant number warranted the exclusion of the missing cases from the multivariable analyses [42] (Table 1).

**Table 1:** Proportion of patients of each characteristic that experience the respective outcome(s)

	Outcome		
	Inpatient admission	72 h revisit	72 h revisit, admitted
Triage priority 1	216 (73.7 %)	9 (12 %)	3 (4 %)
Triage priority 2	1771 (62.2 %)	133 (12.3 %)	62 (5.8 %)
Triage priority 3	4928 (34.7 %)	847 (9.1 %)	309 (3.3 %)
Triage priority 4	419 (19.1 %)	115 (6.5 %)	33 (1.9 %)
Missing priority	14 (16.9 %)	5 (7 %)	2 (3 %)
Age 18–40 years	1881 (25.5 %)	484 (8.8 %)	158 (2.9 %)
Age 40–65 years	2392 (35.5 %)	399 (9.2 %)	144 (3.3 %)
Age 65–80 years	1909 (51.0 %)	174 (9.5 %)	74 (4.0 %)
Age >80 years	1166 (66.3 %)	52 (8.8 %)	33 (5.6 %)
Male	3206 (40.6 %)	522 (11.1 %)	214 (4.6 %)
Female	4142 (35.4 %)	587 (7.8 %)	195 (2.6 %)
Dec–Feb	1832 (37.9 %)	253 (8.4 %)	106 (3.5 %)
Sep–Nov, Mar–May	3687 (37.6 %)	554 (9.0 %)	212 (3.5 %)
Jun–Aug	1829 (36.8 %)	302 (9.6 %)	91 (2.9 %)
Mon	1277 (38.5 %)	179 (8.8 %)	62 (3.0 %)
Tue–Fri	4283 (37.4 %)	643 (9.0 %)	238 (3.3 %)
Sat–Sun	1788 (36.8 %)	287 (9.4 %)	109 (3.6 %)
00:00 – 08:00	1347 (36.7 %)	253 (10.9 %)	97 (4.2 %)
08:00 – 16:00	3280 (36.8 %)	426 (7.6 %)	162 (2.9 %)
16:00–00:00	2721 (38.7 %)	430 (10.0 %)	150 (3.5 %)
≥100 % occupancy	1577 (35.0 %)	245 (8.3 %)	99 (3.4 %)
95–100 % occupancy	2161 (37.7 %)	N/A	N/A
0–95 % occupancy	3610 (38.5 %)	N/A	N/A
<100 % occupancy	N/A	864 (9.3 %)	310 (3.3 %)
Total	7348 (37.5 %)	1109 (9.0 %)	409 (3.3 %)

7,348/19,620 = 37.5 % of cases were admitted to hospital. Crude analyses revealed that the admitted proportion was smaller at times of more pronounced access block: 35.0 % at ≥100 % occupancy, 37.7 % at 95–100 % occupancy, and 38.5 % at <95 % occupancy (*p* < .001). 1,109 (9.0 %) of the 12,272 cases discharged revisited within 72 h. 409 (3.3 %) revisited and were admitted. No significant associations were established between access block and 72 h revisits. EDLOS was more than 20 min longer (3.76 vs. 3.38 h) (*p* = .01) at ≥100 % in-hospital occupancy than at <100 % for patients of triage priority 1–2, while no difference was detected for patients of triage priority 3–4 (*p* = .23) or the total group (*p* = .61) (Table 2). Parametric and non-parametric tests agreed on this point.

**Table 2:** Median EDLOS in relation to occupancy, stratified by triage priority (69 cases missing)

	Triage priority				Total	
	1–2		3–4		(*p* = .61)	
	(*p* = .01)		(*p* = .23)			
Occupancy	<100 %	≥100 %	<100 %	≥100 %	<100 %	≥100 %
	(*N* = 887)	(*N* = 267)	(*N* = 8392)	(*N* = 2657)	(*N* = 9337)	(*N* = 2935)
EDLOS [h] (IQR)	3.38 (2.33–4.85)	3.76 (2.40–5.67)	3.17 (2.17–4.52)	3.08 (2.08–4.59)	3.17 (2.18–4.55)	3.13 (2.10–4.70)

### Adjusted results

Age violated the assumption of linearity in the logit and was transformed to the ordinal scale (18–40, 40–65, 65–80, and >80 years) before inclusion in the multivariable models. Cutoffs were established prior to analysis and relied on perceived clinical relevance. The range of 40–65 years was used as a reference. Hospital occupancy passed the test for linearity and was included in its continuous form in all the multivariable models. Cell counts were below five for <85 % and >105 % occupancy levels, suggesting that the interval in between allowed for the most reliable models. Since Mahalanobis distance, tolerance, and VIF statistics did not indicate any major problems with multicollinearity or multivariable outliers, all models were pursued as planned (Table 3).

**Table 3:** Odds ratio (OR) for outcome per percent change in in-hospital bed occupancy, logistic regression, adjustment sets 1–3

Outcome	Adj. set	Reg. coeff	SE	Wald chi^2^	*p*	OR	95 % CI for OR	
							Lower	Upper
Admission	Step 1	−0.008	0.003	8.612	.003	0.992	0.986	0.997
	R^2^ = 0.00							
	Step 2	−0.008	0.003	6.401	.011	0.992	0.985	0.998
	R^2^ = 0.15							
	Step 3	−0.008	0.003	6.394	.011	0.992	0.986	0.998
	R^2^ = 0.16							
72 h revisit	Step 1	0.005	0.006	0.714	.398	1.005	0.993	1.017
	R^2^ = 0.01							
	Step 2	0.005	0.005	0.683	.409	1.005	0.994	1.015
	R^2^ = 0.01							
	Step 3	0.005	0.005	0.906	.341	1.005	0.995	1.016
	R^2^ = 0.01							
72 h revisit, admitted	Step 1	0.003	0.009	0.099	.753	1.003	0.985	1.022
	R^2^ = 0.00							
	Step 2	0.002	0.008	0.105	.745	1.002	0.988	1.018
	R^2^ = 0.02							
	Step 3	0.003	0.007	0.180	.671	1.003	0.989	1.017
	R^2^ = 0.02							

**Fig. 2:**
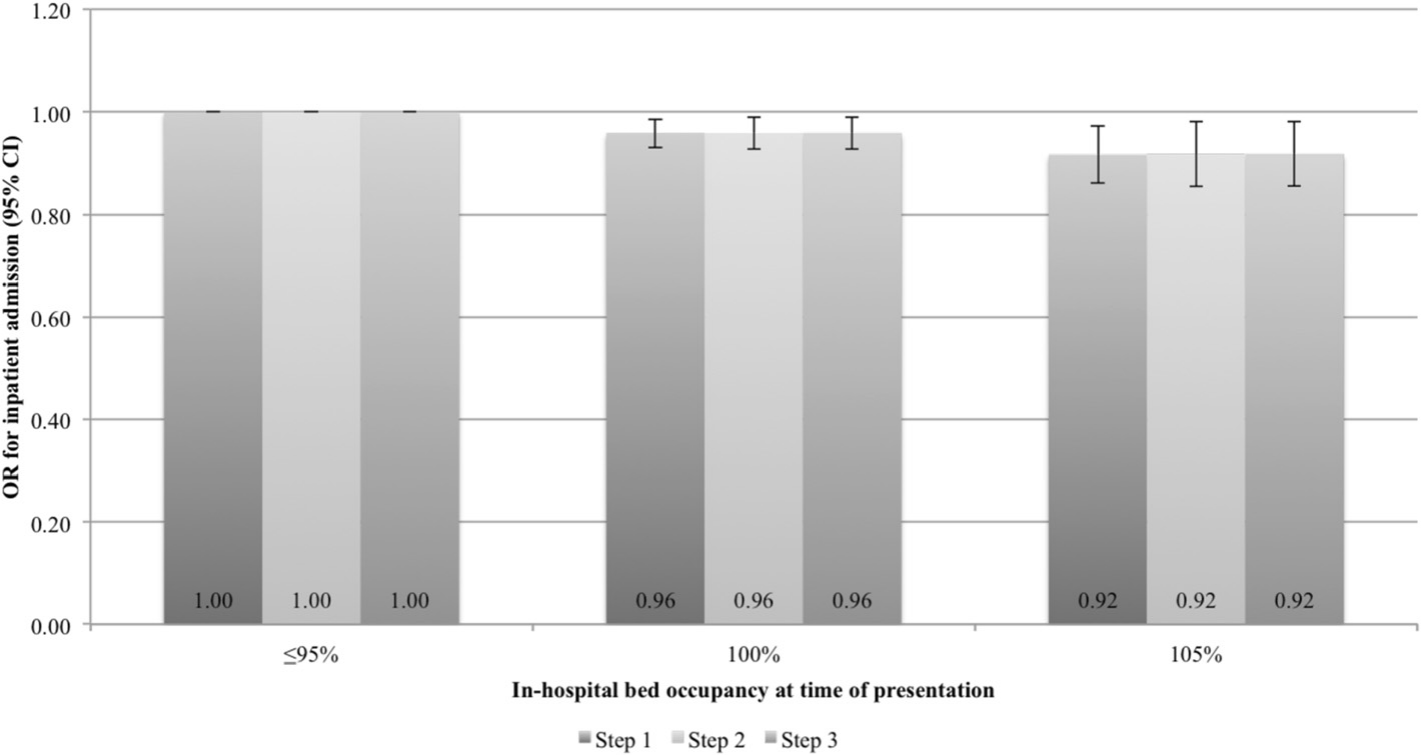
Odds-ratio of inpatient admission at different levels of hospital occupancy, adjustment sets 1–3

The negative association between access block and the likelihood of inpatient admission was significant in the main analysis as well as in both sensitivity analyses, yielding additional support for the results. Table 3 presents an account of the change in odds for admission resulting from a 1 % increase in hospital bed occupancy. The models addressing admission did not suffer from large residuals and predicted a fair portion of events. Meanwhile, models relating access block to 72 h revisits, both resulting in subsequent admission and not, revealed no significant associations (Table 3). These models exhibited some large residuals and had lower explanatory value, indicating that variables not available to us influenced the outcome. The odds ratios (ORs) for hospital admission at 5 % increments relative to 95 % occupancy are displayed in Fig. 2.

## Discussion

The negative association between access block and the probability of inpatient admission supports the hypothesis that ED patients with acute abdominal pain are less likely to be admitted to the hospital at times of access block. The effect appears somewhat attenuated compared to in an undifferentiated ED population [16]. The sample size and power calculations indicate that the study was powered well to detect the pre-specified differences. Moreover, the absence of system-crashes during the study period limits the risk of information bias. The absence of an association to 72 h revisits suggests that discharges from the ED were no less appropriate at times of access block than otherwise. However, this outcome does only capture macro level patterns and says nothing about rare (but disastrous) outcomes such as mortality. The positive association between in-hospital bed occupancy and EDLOS in patients of triage priority 1–2 who were ultimately discharged from the ED could be interpreted as support for the hypothesis of their being subject to more evaluation and/or treatment in the ED at times of access block (reflecting e.g. turnaround times for radiology). However, it could also be indicative of longer waiting times for diagnostics and treatment in the ED. This could be of detriment to patients suffering from time-sensitive conditions.

Since most evaluations of radiology as a means to rule out time-sensitive conditions in abdominal pain have been performed in the hospital setting, strategies where radiology and other outpatient management strategies replace hospital admission need more thorough validation. Future studies should include more granular endpoints (such as mortality) in the patients discharged from the ED, as well as more detailed data about which procedures and interventions were performed in the ED (e.g. radiology), in order to clarify the viability and safety aspects of the observed effect. Such studies would allow for answering the question about whether the observed admission-bias is an expression of increased risk taking in ED staff (by discharging potentially sick patients home) or if a larger proportion of patients receive necessary evaluation and treatment in the ED and that unnecessary inpatient admissions thereby are averted, at times of access block.

Apart from incorporating this perspective, future studies should include more hospitals in order to improve the external validity of the results. This would remedy limitations posed by that the present study only captured revisits to the study site and to the ED at a nearby hospital, but not to other EDs in the region or to primary care. Geographical boundaries (45min by car to the second closest hospital) are likely to somewhat limit the proportion of patients who revisit another ED. International readers may also note that the regional emergency system differs from systems in other countries, mainly in that emergency medicine specialists are scarce and that EDs are subdivided into specialty units staffed by physicians from the inpatient clinics.

## Conclusion

The study findings support the hypothesis that the management strategy in patients with acute abdominal pain changes at times of access block, so that patients are less likely to be admitted to the hospital. The lack of an association between access block and 72 h revisits to the ED does not suggest a corresponding decrease in the appropriateness of discharges, but more granular outcome measures need to be addressed in order to clarify the safety aspects of the observed effect.

## Additional files

Additional file 1: **Causal model for in-hospital admission.** Causal model showing relationships explaining in-hospital admission, in patients with abdominal pain. Diagram was used to identify the minimally sufficient adjustment set for the multivariable models. (PDF 249 kb)

Additional file 2: **Causal model for unplanned 72-h revisits.** Causal model showing relationships explaining 72-h revisits, in patients with abdominal pain who were diverted from the ED at index. Diagram was used to identify the minimally sufficient adjustment set for the multivariable models. (PDF 75 kb)
