# Supplementary figures and images for: Patients presenting at the emergency department with acute abdominal pain are less likely to be admitted to inpatient wards at times of access block: a registry study
Source: Scand J Trauma Resusc Emerg Med. 2015 Oct 7;23:78. doi: 10.1186/s13049-015-0158-3 (PMC4596503; doi:10.1186/s13049-015-0158-3)

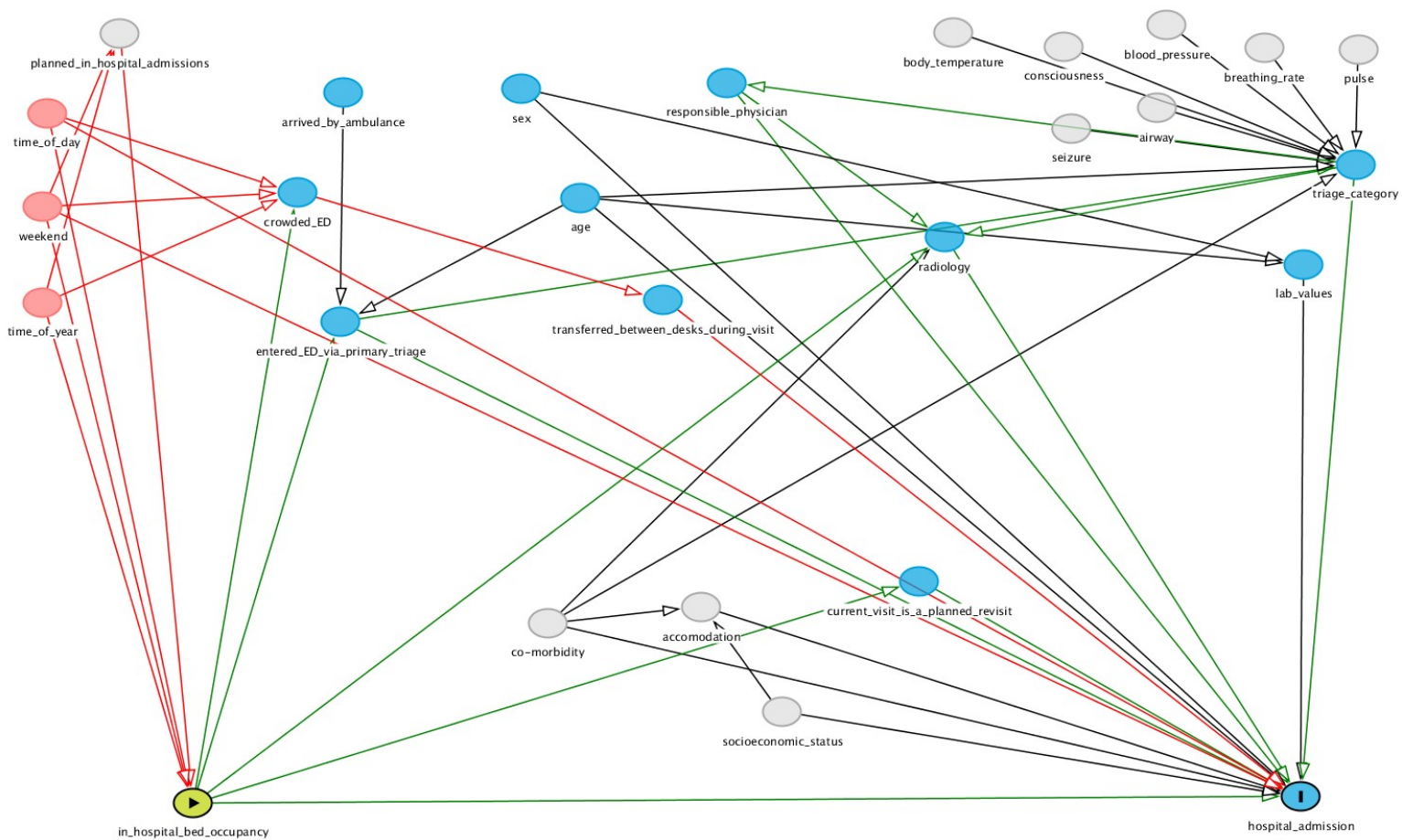

Supplement: Additional file 1: — Causal model for in-hospital admission. Causal model showing relationships explaining in-hospital admission, in patients with abdominal pain. Diagram was used to identify the minimally sufficient adjustment set for the multivariable models. (PDF 249 kb) [file 13049_2015_158_MOESM1_ESM.pdf]

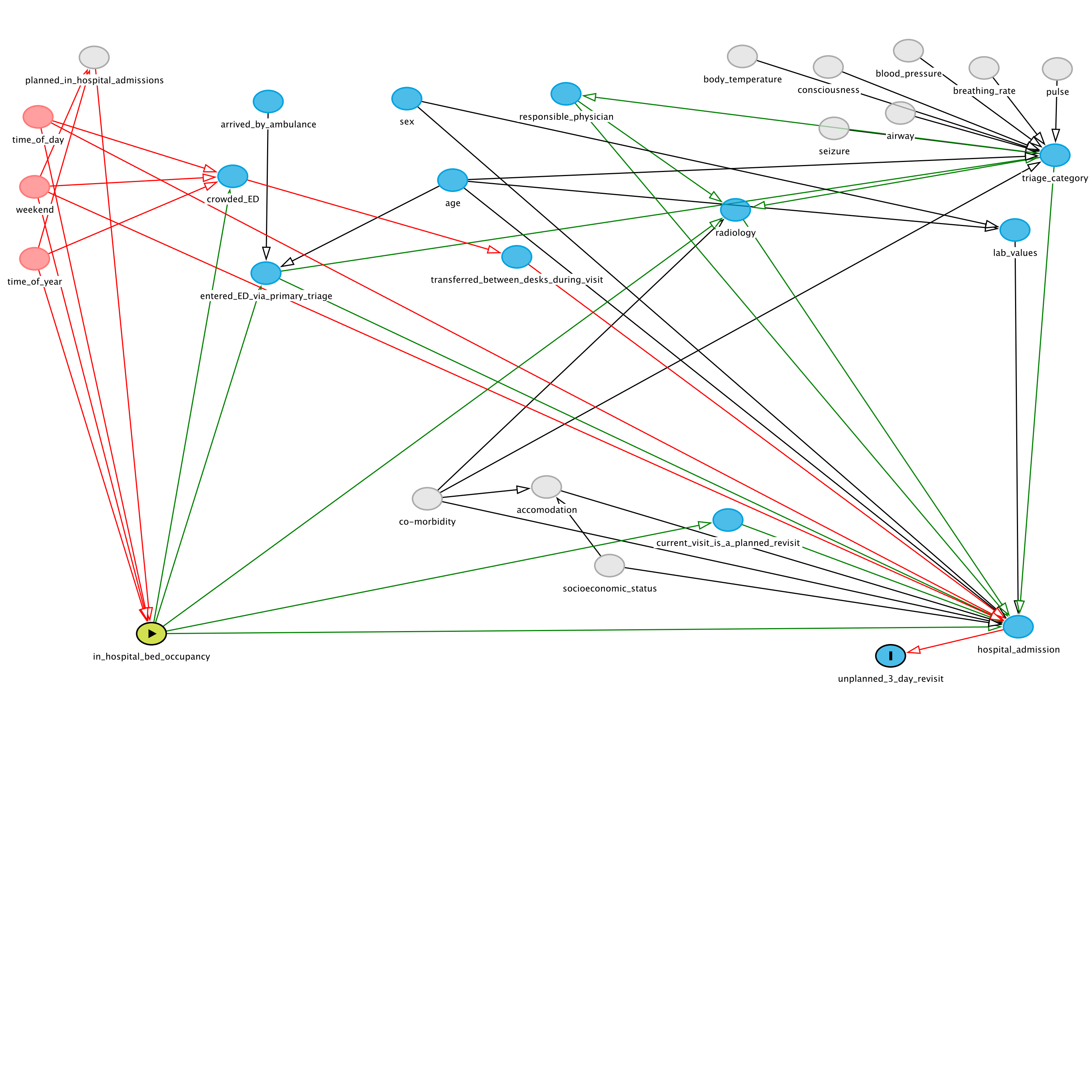

Supplement: Additional file 2: — Causal model for unplanned 72-h revisits. Causal model showing relationships explaining 72-h revisits, in patients with abdominal pain who were diverted from the ED at index. Diagram was used to identify the minimally sufficient adjustment set for the multivariable models. (PDF 75 kb) [file 13049_2015_158_MOESM2_ESM.pdf]
